# Probing the steel-concrete interface microstructure using FIB-SEM nanotomography

**DOI:** 10.1617/s11527-025-02602-3

**Published:** 2025-02-21

**Authors:** Thilo Schmid, Nicolas Ruffray, Michele Griffa, Zhidong Zhang, O. Burkan Isgor, Ueli M. Angst

**Affiliations:** 1 ETH Zürich, Laura-Hezner-Weg 7, 8093 Zürich, Switzerland; 2 Empa, Swiss Federal Laboratories for Materials Science and Technology, ETH-Domain, Überlandstrasse 129, 8600 Dübendorf, Zürich, Switzerland; 3 Oregon State University, Corvallis, OR 97331 USA

**Keywords:** Steel-concrete interface, FIB-SEM nanotomography, Corrosion, Durability, Microstructure

## Abstract

While it is widely accepted that the steel-concrete interface (SCI) plays an important role in governing the long-term durability of reinforced concrete structures, the understanding about the primary features of the SCI that influence corrosion degradation mechanisms has remained elusive. This lack of knowledge can be attributed to, firstly, the complex heterogeneous nature of the SCI, and secondly, the absence of established experimental techniques suitable for studying the relevant SCI features. Here, we use focused ion beam—scanning electron microscopy (FIB-SEM) nanotomography to obtain high-resolution 3D tomograms of the SCI. Five tomograms, spanning volumes ranging from 8000 to ${200\,000}\,{\upmu \hbox {m}^{3}}$, of both non-corroded and corroded SCIs were acquired. The achieved voxel size falls within the range of 30–50 nm, which captures capillary pores highly relevant for moisture and ion transport. Potential pitfalls when applying the FIB-SEM technique to the SCI are highlighted, including aspects related to the electron detectors. We present an image processing pipeline that reduces artifacts and generates tomograms segmented into solid matrix and pore space. Furthermore, to characterize the SCI pore structure, diffusion tortuosity and porosity profiles. The analysis showed that there is a pronounced anisotropy in the pore structure. This work demonstrates that the FIB-SEM technique can be applied to acquire high resolution tomograms of the SCI pore structure, which can be digitally analyzed to inform transport models of the SCI.

**Supplementary Information:**

The online version contains supplementary material available at 10.1617/s11527-025-02602-3.

## Introduction

It is widely recognized that the steel-concrete interface (SCI) plays a crucial role in the long-term performance and durability of reinforced concrete structures [1–3]. Various microstructural features related to the SCI, such as phase assemblage and pore solution chemistry [4–9], pore space structure [10, 11], macroscopic defects [12–18] and interfacial moisture distribution [19–23] have been identified to strongly affect corrosion of the embedded steel. The importance of the interfacial microstructure has been particularly highlighted in recent studies that proposed a model relating the steel corrosion kinetics to the moisture distribution and the microstructure at the SCI [11, 22]. To consider the chemical composition of the pore solution within concrete in space and time, models have been proposed based on solving reactive-transport equations [24–26]. Similarly, models for the prediction of the moisture distribution in concrete have been proposed [27–29]. The aim of these modeling approaches is to better understand pore-scale processes driving steel corrosion in concrete or other concrete degradation mechanisms. However, despite extensive research spanning several decades, the primary SCI characteristics, which govern steel corrosion, are fundamentally still largely unclear [2]. An example of such a process, known from experimental studies but not yet fully understood at the theoretical level, is that corrosion products do not stay where they are produced. Instead, they move and fill pores non-uniformly around the steel [10, 11, 28, 30].

The lack of understanding can be attributed to the complex and heterogeneous nature of the SCI [1]. Additionally, another important reason contributing to the limited understanding and, to some extent, the contradictory findings in the literature [2] is the absence of well-established experimental techniques suitable for studying the SCI and, especially, techniques capable of revealing the features of relevance for the degradation mechanisms affecting the long-term durability of reinforced concrete. In a recent extensive literature review [31], over twenty experimental methods were critically assessed with respect to their aptness for the study of relevant features of the SCI. The review revealed a severe lack of established techniques suitable for characterizing the SCI at a representative scale and with sufficiently high resolution. Representative scales have been identified in the literature to be around ${100}^3 \,{\upmu \hbox {m}^{3}}$ [32–34]. A particularly challenging aspect identified in [31] included the fact that the SCI microstructure is 3D and that 2D imaging techniques, e.g., ex-situ microscopic techniques such as optical or scanning electron microscopy (SEM), are generally not capable of providing such 3D information. This limitation of viewing features only in 2D sectional planes may lead to misinterpretation, for instance, with respect to interconnectivity of pores or the size of features such as quasi-spherical voids. Three-dimensional characterization techniques exist, among which computed X-ray tomography currently seems the most widely used one [17, 35–37], followed by a few applications of neutron tomography or combined neutron and X-ray tomography [18, 38]. While these techniques can reveal interesting 3D features of the SCI microstructure, their limited spatial resolution poses a challenge in resolving the SCI at a scale below approx. 10 micrometers [31]. This limitation applies, in particular, because of constraints about the size of samples that can be retrieved from the steel-concrete interfacial zone. It is extremely challenging, if not impossible, to obtain specimens of the interfacial region that would be small enough for submicron (synchrotron-based) X-ray tomography, without risking significant damage of the features of interest during sample preparation. Another weakness, particularly of X-ray computed tomography, is its sensitivity to imaging artifacts arising from the pronounced differences in X-ray attenuation coefficients of the steel and cementitious matrix, that may give rise to X-ray beam hardening effects of polychromatic X-ray beams [39–42], which in turn may impair the interpretation and analysis of the obtained images.

An alternative imaging technique that is capable of yielding three-dimensional information and at much better spatial resolution than X-ray and neutron tomography is focused ion beam - scanning electron microscopy tomography, generally abbreviated as FIB-SEM nanotomography, or FIB-nt [43, 44]. The approach essentially involves serial milling of sections by means of a FIB to generate stacks of sections with nanometer precision, imaging of each section by SEM, and using these SEM micrographs to create a 3D image with a spatial resolution at the nanometer scale. Another advantage is that the FIB milling typically introduces fewer artifacts than mechanical polishing methods [31]. FIB-SEM nanotomography has been used to study cementitious materials for, e.g., characterizing particle shape and size distribution [43, 44] and their relationships [45] for Portland cement powders. Further studies characterized capillary pores and Hadley grains [46, 47], and distinct hydrated phases [48]. Furthermore, it was used for characterizing the geometrical and topological properties of a high performance concrete’s paste pore space, to estimate transport properties [49]. Preliminary results of an exploratory study using FIB-SEM nanotomography to characterize the SCI were reported in [31].

### Objectives and scope

We consider FIB-SEM nanotomography a particularly powerful and promising technique since it can provide 3D information about the microstructure of the interfacial cementitious matrix, including the distribution of different hydrated and unhydrated cementitious phases, aggregates, and the pore structure in the scale where fundamental durability-relevant processes occur. These processes include capillary condensation and water transport, ion transport, crystallization and precipitation of corrosion products, and poromechanical processes.

In this paper, we illustrate the potential of SCI FIB-SEM nanotomography using two types of specimens: An “non-corroded SCI” that may be representative for situations prior to initiation of corrosion, and a “corroded SCI” that may represent the situation after corrosion initiation, where the characterization of other features, including the presence of rust in the matrix, are of interest in developing fundamental understanding about the SCI.

The objective of this work is to propose a workflow covering all steps from image acquisition using the FIB-SEM technique to post-processing the acquired tomograms, including image correction and segmentation of microstructural features of interest. In this work, we will subsequently focus on the SCI pore structure, and apply computational techniques to characterize the final segmented tomograms in the 3D space, such as by random walk simulations to quantify tortuosity and other algorithm-based approaches for assessing pore size distribution and porosity profiles in *xyz* directions.

While the objective of this work is to set the basis for further research leveraging the SCI tomograms, including the generation of domains of the SCI pore structure for modeling purposes, it is outside the scope of this work to cover these aspects in full detail. However, in Sect. 4.4, future perspectives such as for reactive transport modeling are discussed.

## Materials and methods

### Specimens

Two specimens (photographs provided in the supplementary material) were studied in this work to investigate non-corroded and corroded SCI. Both types come from two previous studies and have distinct curing and exposure histories. Therefore, these specimens were not intended to be compared with each other directly, rather were they selected to independently study two different types of the SCI to illustrate the applicability for both corroded and non-corroded SCIs.

The first specimen, designated “NCI” for “non-corroded interface”, contained a stainless-steel tube (diameter 3 mm, wall thickness 0.25 mm, DIN 1.4301) embedded in mortar matrix produced from ordinary Portland cement (CEM I 42.5N), with a water/cement ratio of 0.5 and a sand/cement ratio of 2 (by weight). The sand was a pure limestone sand with maximum particle size of 2 mm. The purpose of using stainless-steel as reinforcement and a mature Portland cement system was to avoid corrosion at the SCI, and thus to be able to study a “non-corroded SCI”. More information about the specimen production and its history can be found in the original study [42].

The second specimen, designated “CI” for “corroded interface”, is concrete produced with a slag cement (CEM III/B) with a 0.5 and 3 water/binder and sand/binder ratio, respectively. The specimen contains an embedded carbon steel reinforcing steel bar (diameter 6 mm, B500B). The maximum aggregate size was 8 mm. A specimen of size ${15\,\text {cm}\times 5\,\text {cm} \times 4\,\text {cm}}$ was cast with three reinforcing steel bars embedded at cover depth 15 mm from both sides. The entire specimen was fully carbonated and allowed to corrode by exposing it to high humidity as well as cyclic wet and dry conditions. They were ponded with demineralized water for up to 6 cycles, with drying in laboratory climate (approx. 30–40% RH) in-between. More details are provided in the original study (in German) [50]. The purpose of this procedure—namely using carbon steel, slag cement, accelerated carbonation, and exposure to different moisture conditions—was to generate a situation where the steel is corroded under carbonation conditions and with a variable moisture exposure history, thus leading to a certain amount of corrosion products distributed in the cementitious matrix surrounding the steel (“corroded SCI”).

All the experiments described above were performed at indoor laboratory temperature ranging between 20 and ${23}\,^{\circ }\hbox {C}$. Table 1 provides a summary of the properties of the two specimens studied in this research.

**Table 1:** Material specifications of the two types of specimens used

Specimen type	NCI (non-corroded SCI)	CI (corroded SCI)
Binder type	OPC, CEM I 42.5 N	CEM III/B (slag cement)
w/b	0.5	0.5
Aggregate/binder ratio	2	3
Aggregate max. diameter	2 mm	8 mm
Steel type	Austenitic stainless steel (DIN 1.4301)	Carbon steel
Steel geometry	Tube, diameter 3 mm, wall thickness 0.25 mm	Ribbed reinforcing steel, diameter 6 mm
Remarks	Well cured and high degree of hydration of the cementitious matrix	Subjected to accelerated carbonation and variable moisture exposure history

### Specimen preparation for microscopy

To prepare the specimens for microscopy, the specimens were first cut, using a water-cooled diamond circular blade, perpendicular to the reinforcement axis, leaving a thickness of 10–15 mm to match the height limitations of the microscopes used in this study. The edges of the cuts were then trimmed to fit inside 1-inch-diameter silicone moulds used for epoxy vacuum impregnation. After being allowed to dry for at least 48 h in a desiccator under vacuum containing desiccant beads, the specimens were vacuum impregnated using Epo-Tek 301 epoxy resin in a CitoVac vacuum impregnation unit from Struers. After at least 24 h of curing at ambient temperature the samples were progressively polished using different grades of sandpaper (500 and 1000 grit) and diamond paste (9, 3 and ${1}\,{\upmu \hbox {m}}$ average grain size). The specimen cleaning in between each polishing grade was performed in an ethanol ultrasound bath. Cutting, vacuum impregnation and polishing of the specimens were all performed to assure that the axis of the reinforcement was perpendicular to the face that would be later observed in the microscopes. After polishing, the specimens were stored to dry in a desiccator for another 24 h prior to mounting. The specimens were then mounted on 1-inch-diameter aluminium stubs using Conductive Carbon Cement and Silver Paint to assure a proper electrical conductivity between the polished face and the mounting stub. Finally, after allowing the mounting materials to dry for 24 h, the specimens were carbon coated using a Bal-Tec CED 030 carbon evaporator. Carbon coating was preferred over metallic coatings to assure a better Back-Scatter Electron (BSE) imaging of the polished surface.

### FIB-SEM nanotomography

**Fig. 1:**
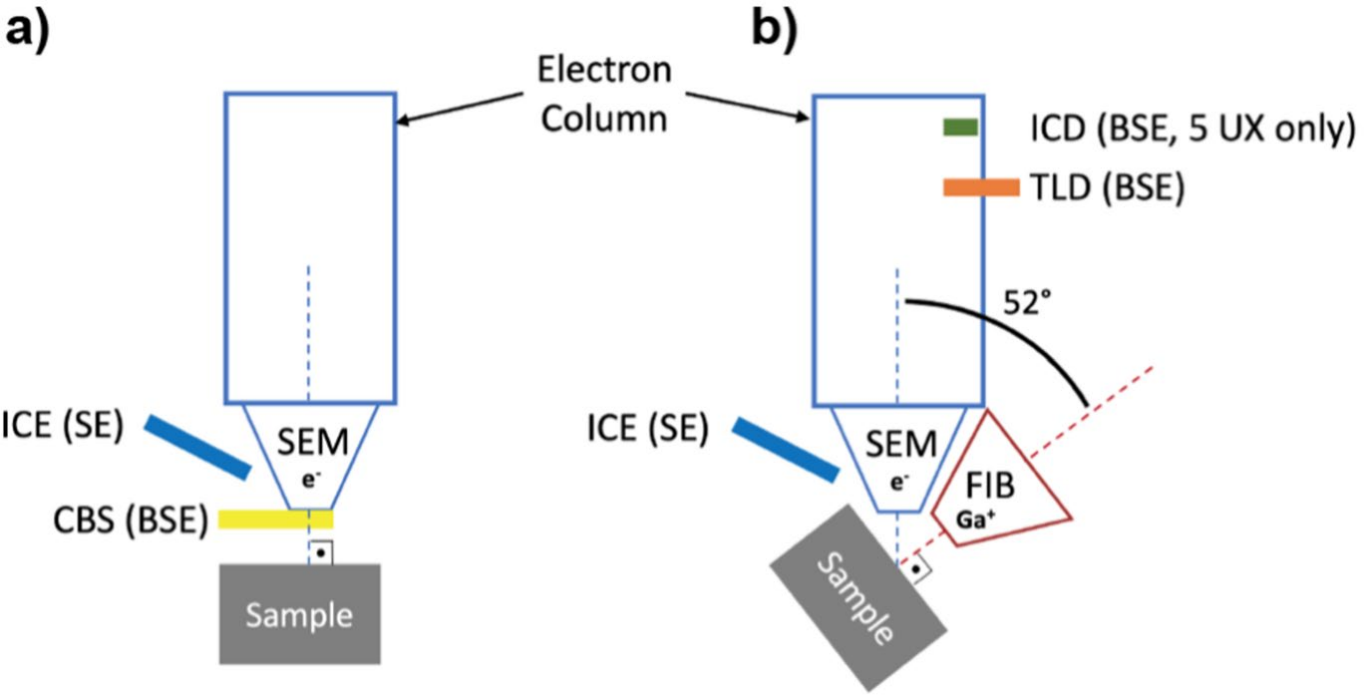
Setup of microscopes with different detectors: Ion Conversion and Electron detector (ICE), Concentric Back-Scatter detector (CBS), Through-Lens Detector (TLD), and In-Column Detector (ICD, only equipped on the 5 UX). **a** Detectors used at ${0}^{\circ }$ tilt in a scanning electron microscope (SEM) with CBS. **b** Detectors used at ${52}^{\circ }$ tilt in a setup combining SEM with a focused ion beam (FIB)

Two different SEMs with similar capabilities and confirmed output performance were used. The Helios 600i from FEI (referred to as 600i in the rest of this article) was used to obtain the tomograms of the NCI specimen, while the Helios 5 UX from Thermo Fisher (referred to as 5 UX) was used on CI type of specimens. Both SEMs were equipped with a secondary Gallium Focused Ion Beam (FIB) positioned at an angle of ${52}^{\circ }$ relative to the electron beam (Fig. 1b). For both microscopes the SEM eucentric and SEM-FIB coincidence points were located at a working distance of 4.1 mm.

Figure 1 presents a schematized layout of the microscopes and the locations of different BSE and Secondary Electron (SE) detectors used during this study. Only the 5 UX was equipped with an In-column Detector (ICD) while other detectors are common to both microscopes. Note that while the Concentric Back-Scatter detector (CBS) produces the most qualitative BSE images, due to its retractable nature, it cannot be used when the sample is tilted at ${52}^{\circ }$ as it would be in the way of the microscope stage.

**Fig. 2:**
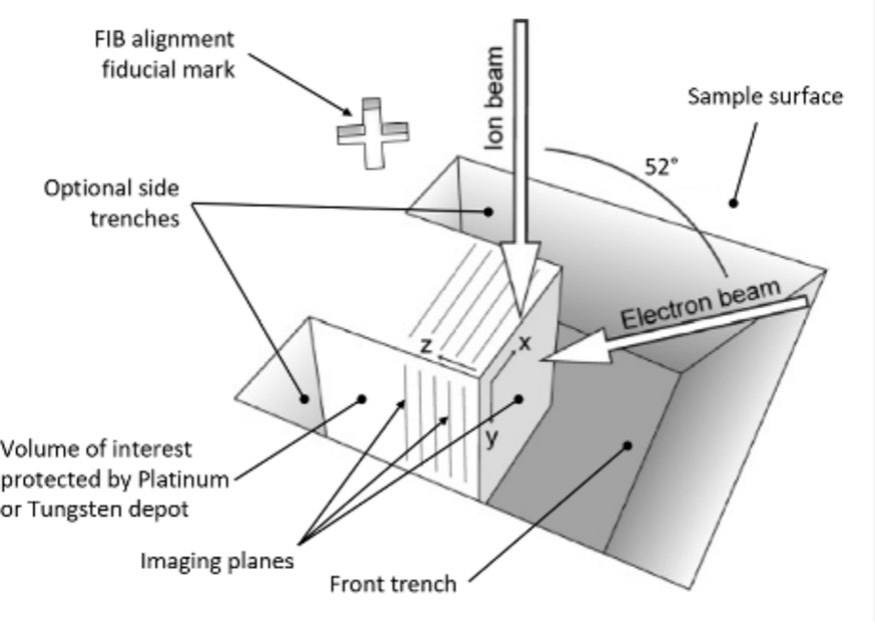
Schematic illustration of the FIB-SEM nanotomography process (adapted from [51])

Figure 2 shows a schematic representation of the FIB-SEM nanotomography preparation and process [51]. First, the polished surface was screened several times along the steel at low magnification (between 150 and 300) prior to making a decision on where to mill the trench for the tomogram. The selected area was then covered by a platinum or tungsten protective layer, using the gas injection system of the microscope in combination with the FIB. The role of this layer was to protect the volume of interest from being damaged by FIB during the tomography preparation and the tomography itself as a FIB image is taken at every tomography iteration for alignment of the ion beam. A trench was opened in front of the volume of interest with the FIB exposing the imaging plane to the electron beam. Note that optional side trenches could also be opened to allow a better evacuation of the milled-away matter and minimize the re-depot growth. A FIB and optional SEM fiducials were engraved to allow beams alignment during the tomography process. In total, the region affected by the microscope including fiducials and side trenches, is about 3 times as wide as the tomogram width. Finally, the imaging plane was polished at low FIB currents to obtain a smooth surface for imaging.

The tomography process was then started and consisted in an automatic iteration of milling away a slice of the volume of interest and taking an image of the exposed plane. The average slice acquisition time was 3–4 min, making the tomography pace 1–2 h per ${\upmu \hbox {m}}$ in the third direction, depending on the voxel size. By repeating this process over all the volume of interest, a stack of images was obtained which can be later used for three-dimensional segmentation and characterization of the microstructure.

The tomograms NCI-3, NCI-4, and CI were acquired over the course of multiple microscopy sessions. The full tomogram was stitched together from up to six smaller ones. This was necessary due to the rather long acquisition times caused by the hardness of the material, leading to long milling times. For instance, the acquisition of the CI tomogram required 116 h (Table 2). As such, the acquisition time is one of the limiting factors for the tomogram’s size.

When not in the microscope, the samples were kept in a glass desiccator with desiccant. Since all the samples had been cured for a long time (several months) before the start of the microscopic investigation, we expect the changes during the scan period or during the storage in the dry environment between measurements to be minimal.

#### FIB milling

All milling operations were performed with the Gallium FIB at a voltage of 30 kV. The opening of the trenches was done with a current of 65 nA. Polishing of the imaging plane was realized with currents of 9.1 and 2.5 nA allowing to obtain an acceptable smoothness for imaging. Tomograms were acquired with a milling current of 9.1 nA and a slice thickness of 30 (NCI-1, CI) or 50 nm (NCI-2, NCI-3).

#### SEM imaging

For general specimen observations and navigation, a high-performance Ion Conversion and Electron (ICE) detector was used at voltages between 3 and 4 kV and currents between 80 pA and 3.2 nA. This SE detector can be used at any tilt angle of the specimen without noticeable loss of image quality. At a ${0}^{\circ }$ tilt angle of the specimen a Concentric Back-Scatter (CBS) detector was used to acquire BSE images to select the acquisition region. The CBS detector was operated at voltages of 5–10 kV and currents of 1.6–3.2 nA. It is important to note that while the CBS detector is able to provide the best quality of BSE images among all available detectors, it cannot be used when the specimen is tilted at ${52}^{\circ }$ for the tomography. This is because the CBS detector is retractable. In its deployed position, the tilted specimen would collide with the detector, as can be observed in Fig. 1.

To acquire BSE tomograms in the tilted position of the specimen, either the Through-Lens (TLD) or the In-Column (ICD) detectors were used. TLD image performed at a voltage of 2 kV, a current of 0.34 nA and a dwell time of ${3}\,\upmu \text {s}$ with a line integration of 8. For the ICD image acquisition, a voltage of 4 kV, a current of 3.2 nA, a dwell time of ${4}\,\upmu \text {s}$ and a line integration of 2 were used. These parameters were empirically obtained to achieve an acceptable compromise between image quality and acquisition time. The horizontal field width (magnification) and the pixel resolution of the images were chosen to obtain a pixel size of either 30 or 50 nm, together with an identical tomography slice thickness. This procedure was selected to achieve isometric voxels. During the tomography acquisitions, both tilt correction and dynamic focus were used to compensate the ${38}^{\circ }$ angle between the electron beam and the imaging plane normal.

To increase the imaging quality of in-column located detectors, such as the TLD and ICD, both microscopes used in this study possess a proprietary technology called immersion mode. When active, this mode allows the detection of a greater number of electrons by using an electromagnetic field applied at the tip of the electron column (see supplementary material for an illustration). It is important to note here that during the tomographic acquisition, the TLD detector requires the usage of such immersion mode to produce exploitable images. While being very beneficial for the quality of the produced images, the immersion mode presents the major disadvantage of not being usable if the specimen contains a large amount of a ferromagnetic metal, as discussed in Sect. 4.2.

### Slice images and resulting tomograms

**Fig. 3:**
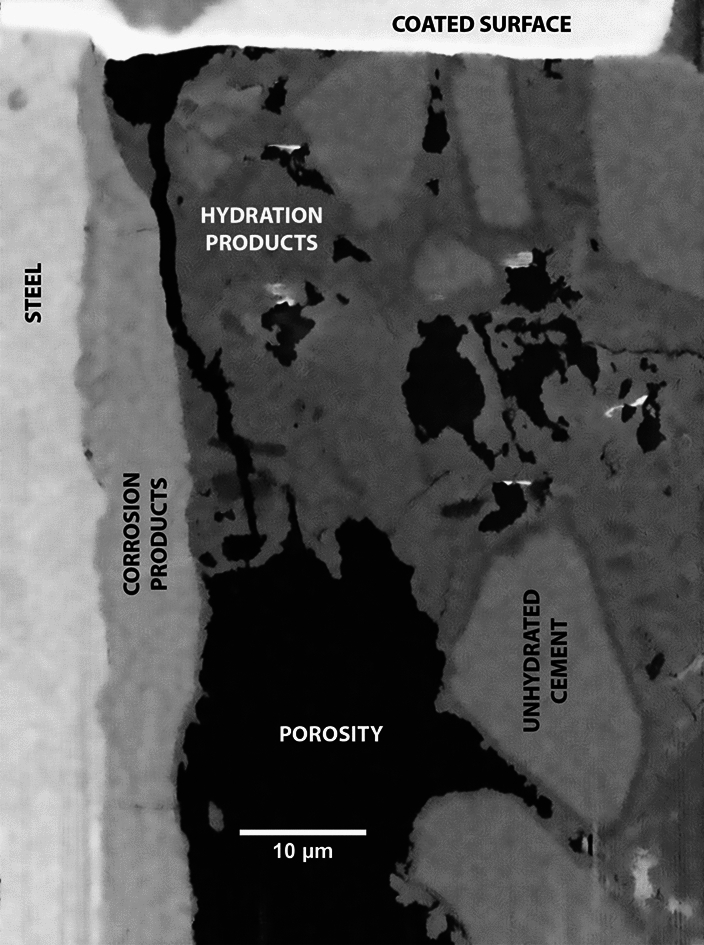
A backscattered electron microscopy slice of the CI tomogram with the constituent phases labeled

The raw data from the microscope consist of a sequence of 2D SEM images, corresponding to the successive slices exposed by FIB milling. An overview of the features of the acquired datasets is presented in Table 2. For each slice, the pixel size is 30 or 50 nm and the pixel values were unsigned integer numbers coded either with a 8-bit depth (for the NCI tomograms) or with a 16-bit one (for CI), corresponding to the binned intensity of the back-scattered electrons incident on the detector. An example image from a CI tomogram is shown in Fig. 3. It shows the SCI with the steel covered by a layer of corrosion products on the left side, and the cementitious matrix to the right. The black regions correspond to epoxy-impregnated pore space; the grey ones correspond to unhydrated cement, which appears slightly brighter, and hydration products more on the darker side, respectively. Fine aggregates also appear in light grey and are difficult to distinguish from unhydrated cement grains.

**Fig. 4:**
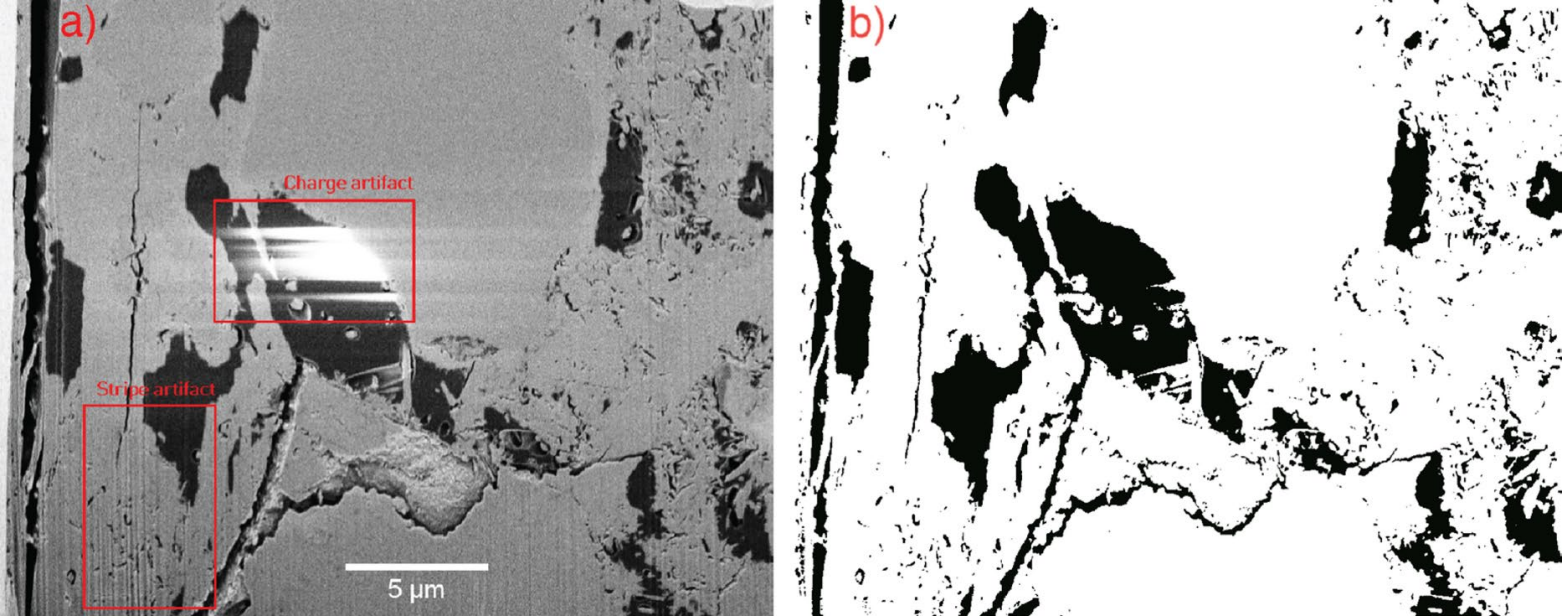
A slice from the NCI-1 tomogram. **a** The original image with the two types of artifacts indicated. **b** Result of the segmentation into pores (black) and solids (white)

### Image artifacts

There are two artifact types in the raw images, which are indicated in Fig. 4a. Charging artifacts originate from buildups of charge in poorly conducting materials, and appear as bright spots of (almost) saturated pixels. This is usually mitigated by a protective Platinum or Palladium layer [52]. In this case, the samples were coated with carbon to keep the possibility of BSE imaging. Unfortunately, there are still numerous charging artifacts present in the NCI tomograms, despite of the applied coating. The charging artifacts differed in size and numbers between the datasets. They were removed manually (Supplementary Figure 3). Another artifact present in some of the images are stripe (also referred to as waterfall, or curtaining) artifacts. They are caused by different ion milling rates between constituent phases and result in vertical stripes. Illustrations of these artifacts and their treatment are shown in Supplementary Figures 3 and 4.

### Image processing

**Fig. 5:**
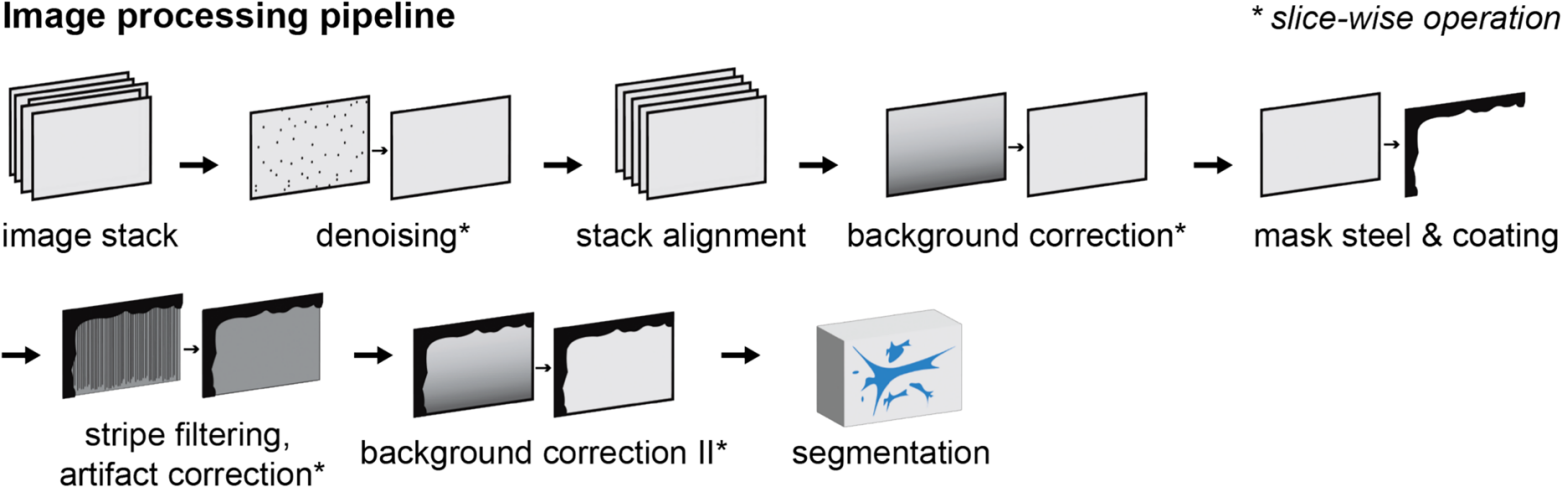
The image processing workflow. The asterisk (*) indicates slice-wise operations, which acted on the two-dimensional cross-sectional images instead of the whole three-dimensional tomogram

The raw tomograms underwent various processing steps, which addressed the aforementioned artifacts and prepared them for the segmentation. Figure 5 provides an overview of the image processing workflow, which was adapted from Münch and Griffa [53]. The individual steps are explained in the following.

#### Denoising

**Fig. 6:**
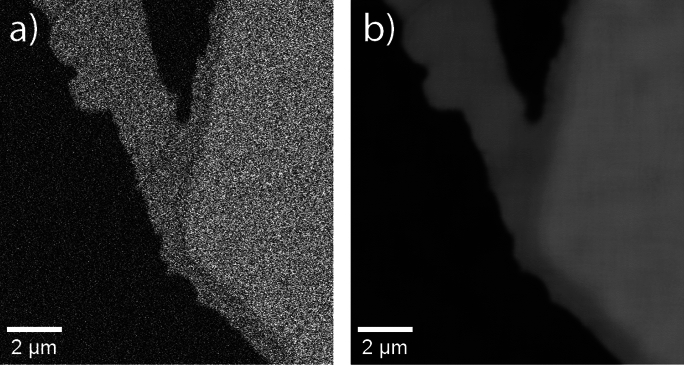
A slice of the CI tomogram before (**a**) and after (**b**) applying the non-local means filter

The tomograms suffered from various levels of uncorrelated noises. The CI tomogram, which was acquired using the alternative in-column detector in the Helios 5UX microscope, is particularly affected. To attenuate the noise, the non-local means algorithm [54, 55] implemented by the ImageJ plugin *DenoisEM* [56] was used. The implementation in this plugin operates on each slice individually and substitutes a given pixel value with the average value of pixels in a certain search window, the average being weighted by the similarity of the two pixel’s neighborhoods. Compared to similar noise reduction procedures, this algorithm excels at preserving edges in the image, which is crucial for the segmentation. The effect of the noise reduction is showcased in Fig. 6.

#### Alignment

Small misalignments of successive images were corrected by using the SIFT (scale-invariant feature transform) algorithm to compute the required translation of each slice to produce an aligned stack [57].

#### Background correction

The geometry of the trench and the tilt of the electron beam led to a vertical gradient in intensity, with the lower parts of the image being slightly darker. This was corrected by modeling the background as a polynomial function, which was subtracted from the original image. Such polynomial function was estimated with an algorithm belonging to the Xlib set of ImageJ plugins by B. Münch [58]. The first background correction facilitates the detection of the steel and the coated surface on the top of the image. This is necessary for creating a mask of these phases in the following step. After the stripe filtering (see below), the background was computed and subtracted once more, this time only accounting for the (masked) volume of interest, namely the mortar or concrete and the pore space.

#### Masking of the steel and coated surface

After the first background correction, a binary mask of the steel, the coated surface, and corrosion products was created. They appear brighter than the remaining solid constituents, and could therefore be detected by manually specifying a voxel value range as well as a number of points in the steel and the coated surface regions, acting as seed points for a segmentation procedure based on the 3D region growing algorithm also implemented in the Xlib ImageJ plugins set [59]. Such region growing algorithm identified all the voxels with value in such range, thus belonging to these phases, each voxel being connected to at least one other already belonging to such class. In the subsequent steps, the classified (or “masked”) voxels were ignored.

#### Stripe filtering

To reduce the stripe artifacts, a combined wavelet-FFT filter [60], also implemented in the Xlib ImageJ plugins set, was used. The algorithm depends on three main parameters: the choice of wavelet base (in this case, only Daubechies wavelets were considered), the damping coefficient $\sigma$, and the decomposition level $\ell$. Each parameter has been varied independently and the optimal combination has been selected by visual assessment. The best results have been achieved by using the DB15 wavelet base with the parameters $\sigma =8$ and $\ell =2$. It was observed that higher decomposition levels $\ell$ resulted in thin vertical pores being affected. Therefore, a relatively low $\ell$ was selected. An example is shown in Supplementary Figure 4.

#### Segmentation

This segmentation step assigned all voxels of the cementitious specimen to distinct categories, corresponding to the constituent phases. Ideally, the categories are steel, corrosion products, pore space, fine aggregates, hydrated cement paste regions, and unhydrated cement ones. However, the contrast offered by the through-lens detector was insufficient to allow multi-phase segmentation for the NCI tomograms. Such phases segmentation was possible only for the CI tomogram, due to the use of the in-column detector offered by the Helios 5UX microscope model. For a better comparison between the tomograms, the discussion will focus on the binary segmentation, i.e., the identification only of pore space and solid phases. The best segmentation results were achieved by using the ImageJ plugin *Trainable WEKA segmentation 3D* [61], a supervised machine learning-based segmentation tool, which was trained on an annotated subset of images. As an unsupervised learning alternative, *Lloyd’s k-means clustering* algorithm [62] also produced satisfactory output on most images.

### Characterization of the SCI pore structure

There are numerous applications for these segmented tomograms. For example, an algorithm-based analysis of the pore space of the imaged specimen, such as the computation of the pore size distribution, or the computation of local porosity profiles. Furthermore, they can be turned into a discretized domain such as a finite elements mesh for reactive transport modeling at the pore scale, like the diffusion of ions and their interactions with the solid phases (e.g. chloride binding).

#### Tortuosity estimation by random walk simulations

Together with the porosity, the tortuosity $\tau$ is one of the characteristic quantities of porous media. There are various definitions of tortuosity [63]. In simple terms, it can be considered the factor between the effective path length and the (euclidean) distance between two points in the pore space, where the effective path always falls within the pore space. The possibilities to compute the tortuosity include random walk-based methods, path-searching methods, and approaches based on skeletonization of the pore space [63]. To illustrate the concept of computing the tortuosity using the segmented tomograms, a random walk method to compute $\tau$ (sometimes referred to as the diffusion tortuosity, $\tau _D$) was used [64]. It relies on the relation

1 $$\begin{aligned} \frac{1}{\tau _D} = \lim _{t\rightarrow \infty } \frac{d}{dt} \langle r^2(t) \rangle \end{aligned}$$

where $r^2(t)$ stands for the displacement squared of the random walker, and *t* denotes the time (i.e. the number of time steps). The angle brackets $\langle ~ \rangle$ denote the ensemble average over all random walkers. In this case, 20 000 random walks were performed, with each random walker taking 20 000 steps. The euclidean displacement from the randomly selected starting position $r(t_0)$ of a walker is regarded as a function of time (i.e. the number of time steps). For each value of *t*, the ensemble average of the displacement is used. The asymptotic slope at high *t* of the averaged squared displacement, plotted against *t*, is identified as the inverse $\tau _D$, in accordance with Eq. 1. Our implementation made use of the open-source Python package *pytrax* [65].

#### Pore size distribution

The pore size distribution (PSD) is an important characteristic of porous materials, which can be obtained in various ways. For pores that can be resolved, there exist multiple definitions of the pore size distribution [66]. The discrete pore size distribution can be computed by dividing the pore space into individual pores—for instance using a watershed algorithm—and assigning them the radius of a sphere of equal volume as the pore size. Here, the continuous pore size distribution was considered. The continuous PSD assigns every voxel the radius of the largest sphere which fits inside the pore space and contains that specific voxel. The resulting pore size distribution is simply the distribution of those voxel values. For that purpose, the ImageJ plugin “Pore size distribution” from the *Xlib* set of plugins developed by Beat Münch [58] was used.

#### Local porosity profiles

**Fig. 7:**
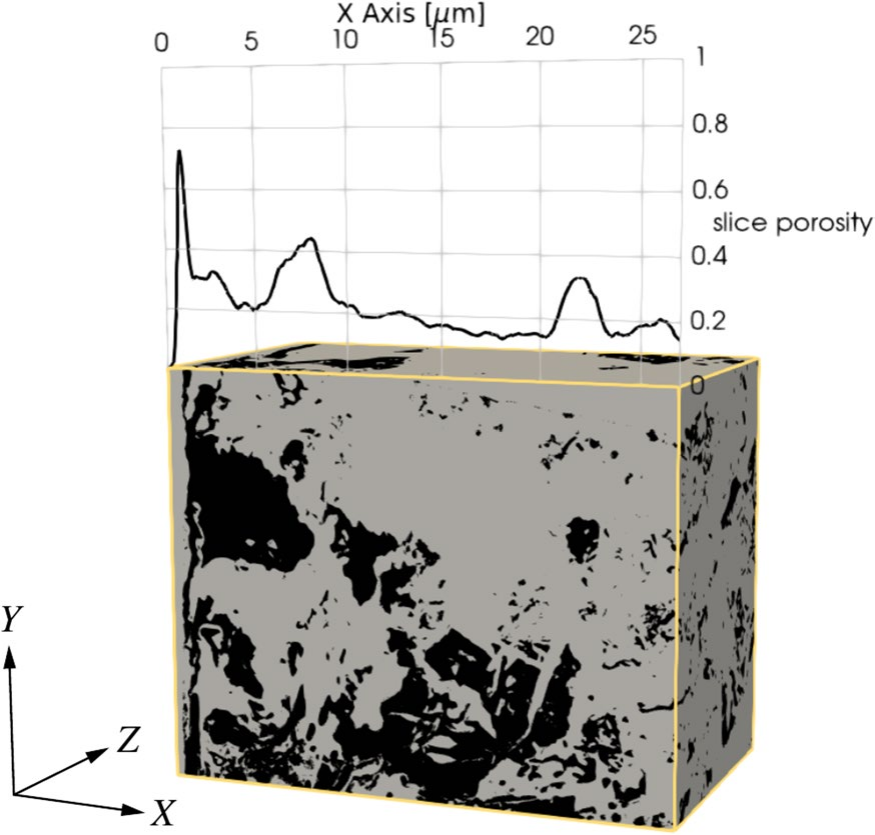
Local porosity profile of the segmented NCI-1 tomogram. It shows the average local porosity in the *YZ*-planes, for all values of *X*

One first relevant pore space characteristic is the local porosity spatial variation. To quantify how the local porosity changes along the three axes—*X*, *Y*, and *Z*—its average value over planar cross-sections, respectively perpendicular to these axes, is computed. The implementation was done in Python and Fiji [67]. The idea is illustrated in Fig. 7 for the *X*-axis of the NCI-1 tomogram. The plotted porosity is the average local porosity computed over the slice in the *YZ*-plane, at that value of *X* where the plane is located. Note that the porosity profile along the *X*-axis shows the porosity as a function of the distance to the steel. This axis is of particular interest because a dependence of the local porosity on the distance to the steel would imply that the mortar’s pore space at the SCI is, statistically, not isotropically homogeneous.

## Results

### FIB-SEM tomograms

**Fig. 8:**
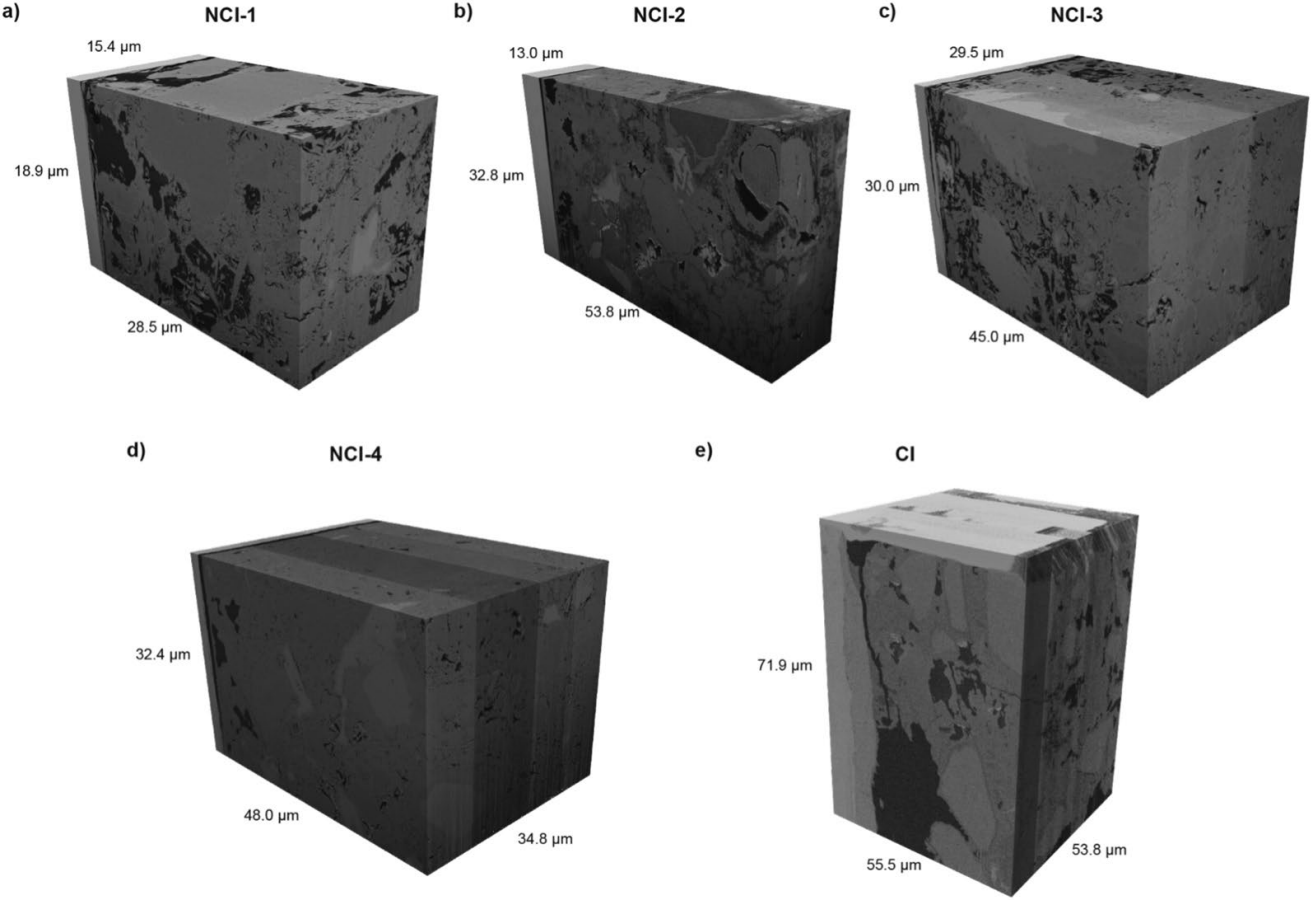
Three-dimensional renderings of the tomograms of the four samples representing a non-corroded interface (NCI) (**a**, **b**, **c**, **d**) and the sample representing the corroded interface (CI) (**e**)

Five tomograms were generated during this study - four for the NCI specimen, and one for the CI specimen. Figure 8 shows three-dimensional reconstructions from the tomograms. Note that the tomograms c), d), and e) in Fig. 8 are composed of blocks exhibiting different average voxel values. These variations stem from the fact that these “large” tomograms (cf. Table 2) were stitched together from several sub-tomograms that were acquired on the microscopes throughout several runs. This is due to limited availability of the imaging equipment, which was generally limited to half days and weekends, combined with the long measuring times. As a result, the volumes of the size of interest could only be acquired over several interrupted runs. As an example, for the tomogram CI, 8 microscopy sessions were needed with a total of approximately 116 h of acquisition time, and additional 20–25 h of sample preparation on the microscope prior to the FIB-SEM nanotomography measurements. At the beginning of each run, the brightness and contrast were adjusted manually such that the first image of the new run looked as similar as possible to the last image of the previous run. Another influencing factor causing the differences in voxel values during acquisition were drifts in brightness and contrast related to the devices, which, in this study, was found to be particularly pronounced on the 5UX instrument, but also occurring on the 600i device, although to a smaller extent. For these reasons, it is challenging to ensure constant image brightness and contrast during acquisition. Thus, these features must be corrected for during subsequent image treatment.

**Fig. 9:**
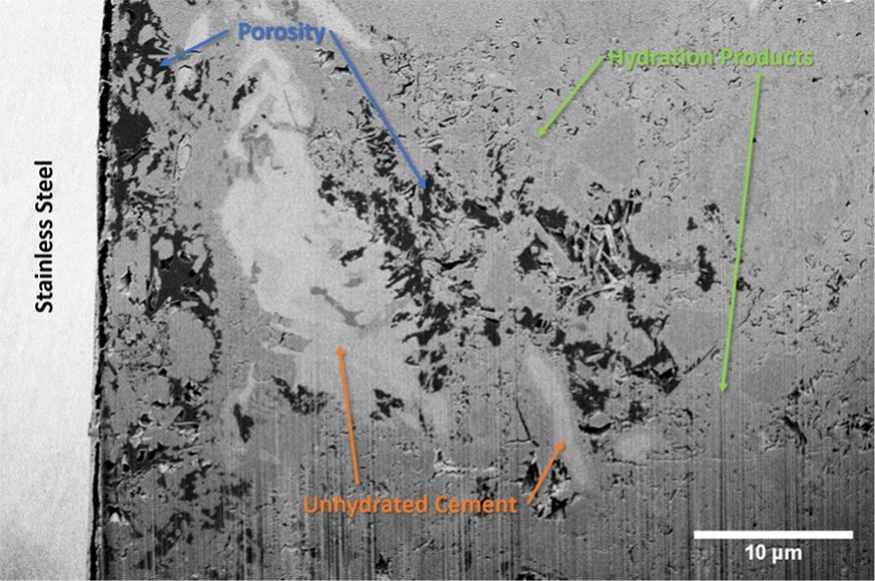
Tomogram slice (SEM imaged section) of specimen NCI-3. Obtained with the Helios 600i (TLD, Immersion Mode, 2 kV, 0.34 nA, ${52}^{\circ }$ tilt). The curtaining effect observed at the bottom of the image comes from the relatively important depth of the tomogram and the presence of pores space that was not filled with epoxy resin, therefore creating a hardness inhomogeneity resulting in this artifact

A typical tomography slice (SEM imaged section) can be observed in Fig. 9. On this image of NCI-3 obtained by TLD detector in immersion mode, the bright vertical strip on the left is the stainless-steel tube while the rest of the image shows the adjacent mortar microstructure. Cement hydrates appear darker, whereas unhydrated cement are lighter (higher voxel values). Epoxy resin filled pore space appears black.

From this image, one can observe the complex structure of pore space present at the steel-mortar interface as well as in the nearby transition zone. The choice of the voxel size is important to be able to observe and later segment small features such as capillary pores. Indeed, derived from to the Nyquist sampling theorem [68, 69], it is considered that a feature must be covered by at least 2.3 adjacent pixels to be segmented as such. Therefore, with this ratio conservatively assumed to be 3 to account for additional uncertainties, the obtained tomograms present a feature resolution of 150 and 90 nm for 50 and 30 nm voxel sizes, respectively. Upon 3D reconstruction and segmentation, the tomography data can provide detailed and interesting information on parameters such as pore size distribution, geometry, interconnectivity, and tortuosity.

**Fig. 10:**
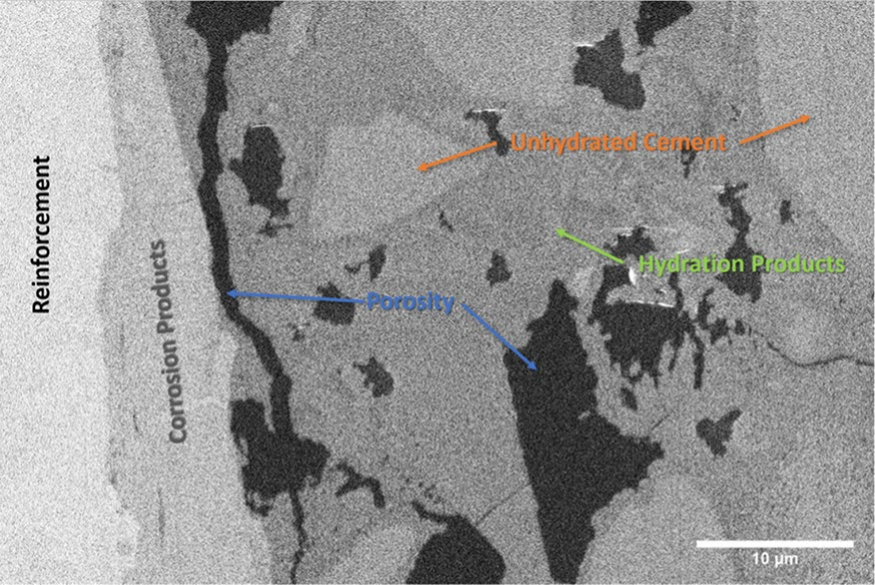
Tomogram slice (SEM imaged section) of specimen CI. Obtained on 5 UX (ICD, Normal Mode, 4 kV, 3.2 nA, ${52}^{\circ }$ tilt)

Figure 10 shows a tomogram slice of specimen CI, obtained by ICD detector in normal mode. Like in Fig. 9, the bright vertical area on the left of the image represents the edge of the reinforcement. Adjacent to this zone, one can observe a slightly darker one which in this case corresponds to corrosion products. By comparing Figs. 9 and 10, it is also possible to notice some differences linked to the use of different detectors. TLD in immersion mode tends to produce sharp images with a good contrast range while ICD in normal mode shows a certain amount of noise. However, the higher level of noise of the ICD can be mitigated during the image processing prior to segmentation.

**Fig. 11:**
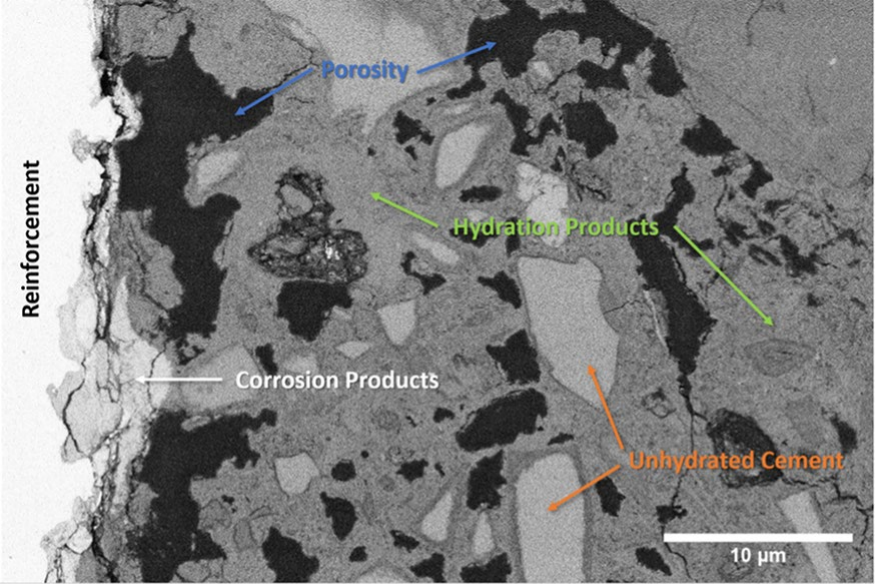
Surface image of SCI of sample CI. Obtained at the sample surface where the tomography trench was opened, taken on 5 UX (CBS, Normal Mode, 4 kV, 3.2 nA, ${0}^{\circ }$ tilt)

Figure 11 shows an image of a physically polished (see section 2.2) surface of the CI specimen at a random location of the SCI obtained by the CBS detector in normal mode at ${0}^{\circ }$ tilt. This image can be compared to the tomogram slice presented in Fig. 10, which was a FIB-polished surface and taken at ${52}^{\circ }$ tilt. Both the physically and FIB-polished image show similarities in microstructure. The presence of corrosion products at the SCI and a visually comparable pore size and distribution indicate that the microstructure observed during the tomography is representative of the one that would be obtained on a physically polished surface of the specimen at ${0}^{\circ }$ tilt

### SCI pore structure characterization

From the five acquired and segmented tomograms, four were used for the algorithm-based analysis of their pore structure: NCI-1, NCI-2, NCI-3, and CI. The NCI-4 tomogram was disregarded due to the absence of a large connected pore cluster, making it unusable for the random walk technique and of lesser interest for future transport simulations. Both the raw tomograms [70] and the segmentations [71] are made available open-access to the research community. Table 2 summarizes their properties, including the overall porosity, i.e., the ratio of voxels classified as pore space to the total number of voxels of the tomogram. Note that the voxel size differs between the four tomograms. The tomogram volume of CI is significantly larger than that of the NCI tomograms. Inevitably, the larger size came at the cost of the longest acquisition time of over 100 h, divided over six sessions. The specimen CI also has the highest porosity, largely due to a large pore close to the steel next to the corrosion products.

**Table 2:** The analyzed tomograms

	NCI-1	NCI-2	NCI-3	CI
Binder type	OPC, CEM I 42.5 N	OPC, CEM I 42.5 N	OPC, CEM I 42.5 N	CEM III/B (slag cement)
Steel type	Stainless steel	Stainless steel	Stainless steel	Carbon steel
Acquisition time (h)	35	18	40	116
Voxel size	$\left( 30\, \textrm{nm}\right) ^3$	$\left( 50\, \textrm{nm}\right) ^3$	$\left( 50\, \textrm{nm}\right) ^3$	$\left( 30\, \textrm{nm}\right) ^3$
Volume (voxels)	$896 \times 711 \times 514$	$985 \times 721 \times 261$	$977 \times 813 \times 607$	$1825 \times 2282 \times 1795$
Side length (${\upmu \hbox {m}}$)	$26.8\times 21.3\times 15.4$	$49.2\times 36\times 13$	$48.8 \times 40.6\times 30.3$	$54.7 \times 68.4 \times 53.8$
Volume (${\upmu \hbox {m}^{3}}$)	9 545	23 117	54 502	232 072
Porosity	23.1 %	9.85 %	10.8 %	31.5 %
Diff. tortuosity $\tau _D$	2.23	2.19	5.19	2.4

**Fig. 12:**
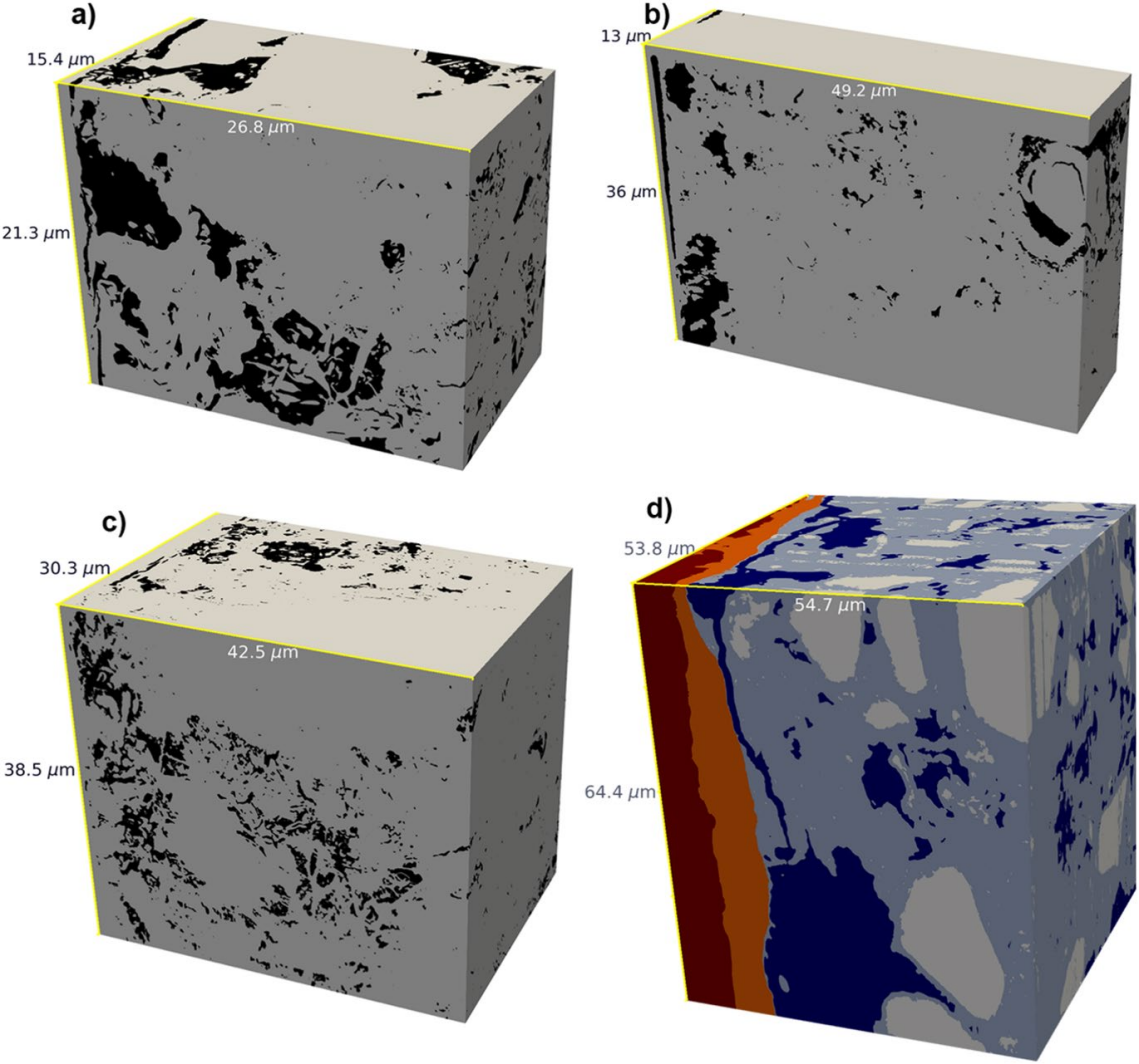
**a**, **b**, **c** Volume rendering of the segmented tomograms NCI-1, NCI-2, and NCI-3, respectively. The segmented phases are pore space (black), and the various solid phases (grey). The SCI is at the back left side. **d** Volume rendering of the segmented tomogram CI. It shows the steel (dark red), corrosion products on the steel (orange), unhydrated cement and aggregates (white), hydrated cementitious matrix (light blue), and pore space (dark blue). Note that the renderings of NCI-3 and CI have been cropped slightly to produce a better image, therefore their size is smaller compared to the values in Table 2

The volume is given in terms of $x \times y \times z$. Volume renderings are shown in Fig. 12. The raw and segmented tomograms [70, 71] are available for download

For the NCI tomograms, the segmentation considered two classes, pore space and solid cementitious matrix. For the CI tomogram, multi-phase segmentation was done—the solids were further divided into corrosion products, hydration products, as well as unhydrated cement and aggregates. The segmented tomogram NCI-1 is shown in Fig. 12a). It is the tomogram with the highest overall porosity among the NCI (mortar) tomograms. Its largest connected pore cluster constitutes more than 90% of the overall pore space. The segmented tomogram NCI-2 (Fig. 12b) is stitched together from four individual tomograms. The raw data was relatively noisy and contained striping artifacts. Certain pores hidden by artifacts might therefore not be reconstructed accurately, although the number of affected pores is relatively low. In contrast to the NCI-1 tomogram, the pore space is in the form of few unconnected pore clusters. Its largest pore is adjacent to the steel surface. Figure 12c) shows the segmented tomogram NCI-3. Like NCI-1, it was only possible to clearly identify two phases. Compared to the other NCI tomograms, the porosity is similar to NCI-2. However, there is a large region with no resolved pores (the lower parts in Fig. 12c), with the rest being similar to NCI-1. There is again a percolating pore cluster at the scale of resolvable pores.

The segmented tomogram CI is depicted in Fig. 12d). It consists of six smaller tomograms from individual microscopy sessions stitched together. On the left, it shows a layer of corrosion products on the steel with no apparent pores at this resolution. Both the steel and the corrosion products were masked during the image processing. As in the other tomograms, there is a large pore next to the steel and corrosion products. Due to this large pore, the overall porosity in the tomogram is rather high. In the middle of the top and right face of the cuboid, there are noticeable shifts in the brightness and contrast of the tomogram, which were corrected (see Sect. 3.1). The phase distribution of the CI tomogram is pores (20.3%), hydrated cement (47%), unhydrated cement (11.9%), corrosion products (17%) and steel (3.8%).

A shared feature of the tomograms is the gap between the steel and the mortar, which was classified as pore space. In some cases, for example in Fig. 3, the walls of the gap are parallel. Therefore, the gap was likely caused by the cutting of the specimen [42], and should be interpreted as an artifact of the sample preparation. The most pronounced gap is found in the NCI-1 dataset, as seen on the porosity profile in Fig. 7a). For the others, there are smaller gap-like pores close to the steel surface (left side of the cuboids in Fig. 12).

## Discussion

### Phenomenological features of the SCI

Across all tomograms, the first ${5}\,{\upmu \hbox {m}}$ of mortar around the steel surface are significantly more porous compared to the pore structure at larger distances. From the perspective of studying corrosion-related transport processes, this is highly relevant.

#### Pore size distribution

**Fig. 13:**
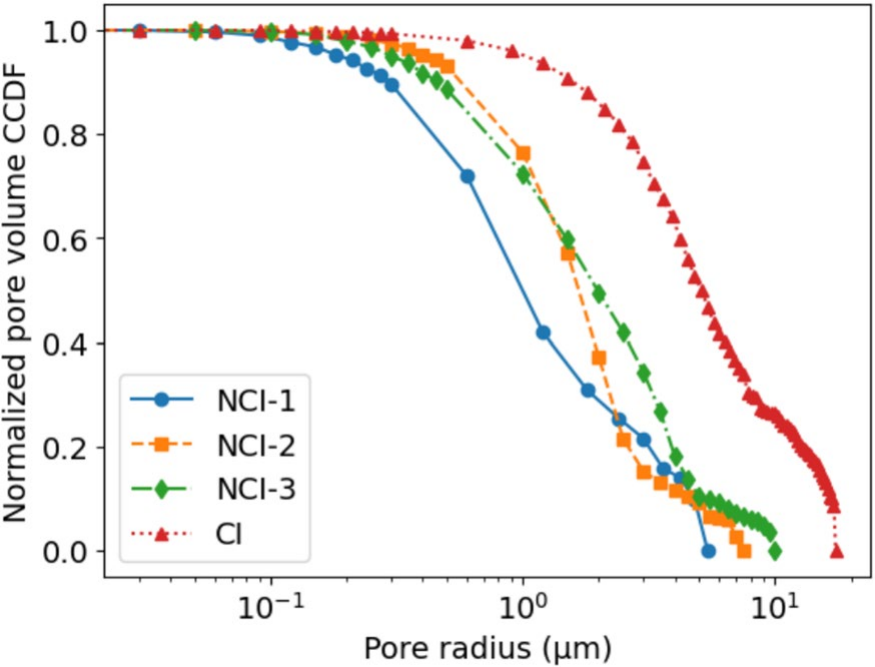
Continuous pore size distribution (PSD) of the segmented tomograms

Figure 13 shows the complementary cumulative distribution function (ccdf) of the continuous pore size distribution computed from the segmented tomograms. The ccdf is a common way to represent the PSD. The values on the y-axis denote how much of the pore space is filled by pores larger than the corresponding value on the x-axis. Comparable PSD data obtained by mercury intrusion porosimetry (MIP) are, for such SCI regions as imaged in this work, not available in the literature, considering the difficulty of preparing specimens encompassing only the SCI. However, even if such data existed, MIP results would likely differ in certain aspects. In contrast to computing the PSD from a tomogram, MIP produces the size distribution of pores accessible to mercury that is forced into the material under pressure. As such, it is well-known that MIP suffers from a so-called ink bottle effect, referring to pores that lie behind narrow pathways. Such pores are reached by the intruding mercury only at intrusion pressures larger than their actual equivalent pore radius value, as estimated by the Lucas-Washburn equation. It follows that MIP-based PSD systematically overestimate the fraction of smaller pores. [72]. While for the image-based approach the ink bottle effect does not exist, its drawback is the limited resolution of the images, leading to very narrow pores—in our case pores with diameters below around 90 nm—to be excluded. [66].

**Fig. 14:**
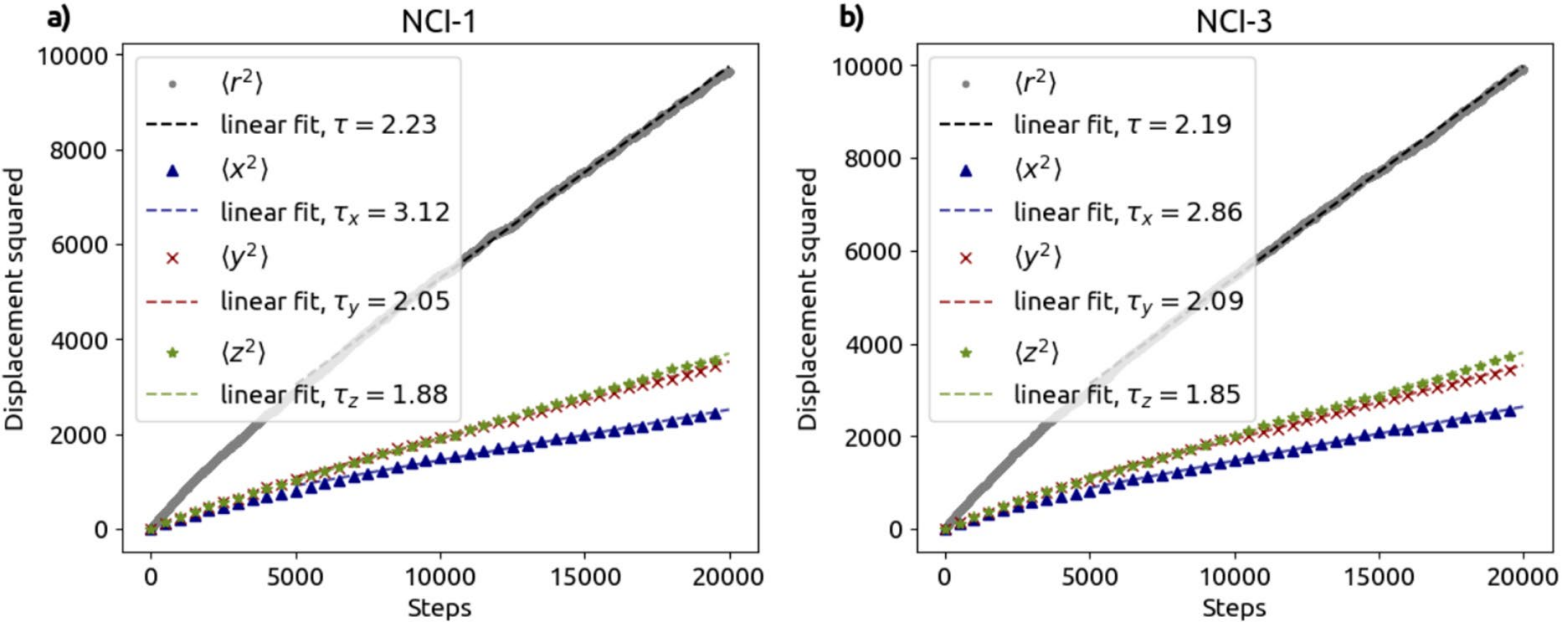
Results of 20 000 random walks in the NCI-1 (**a**) and NCI-3 (**b**) tomograms

#### Tortuosity

For estimating the diffusion tortuosity $\tau _D$ of the segmented tomograms, 20 000 random walkers were simulated. Figure 14 shows the relation between the (ensemble average of the) square displacement of the random walker from its randomly chosen starting position and the number of steps taken. The numbers of walkers and steps were chosen by assessing whether the asymptotic behavior shown in Fig. 14 is well captured by a linear fit. For NCI-1, NCI-2, NCI-3 and CI, the computed $\tau _D$ values derived according to Eq. 1 were 2.23, 2.19, 5.19, and 2.4, respectively. The lower values for NCI-1, NCI-2 and CI are likely due to the porosity being dominated by a single relatively larger pore resulting in more unhindered paths inside the large pore (note that in free space, the tortuosity would be equal to 1).

Due to the absence of comparable studies based on tomograms with a similar resolution, comparing values for $\tau _D$ computed using this method with the existing literature is difficult. However, studies have reported diffusion tortuosities ranging from 3 to 9 for cement paste imaged by X-ray CT with a voxel size of 500 nm [64], and $\tau _D$ values from 4 to 8 for a similar analysis of alkali-activated binders [73].

#### Porosity profiles

**Fig. 15:**
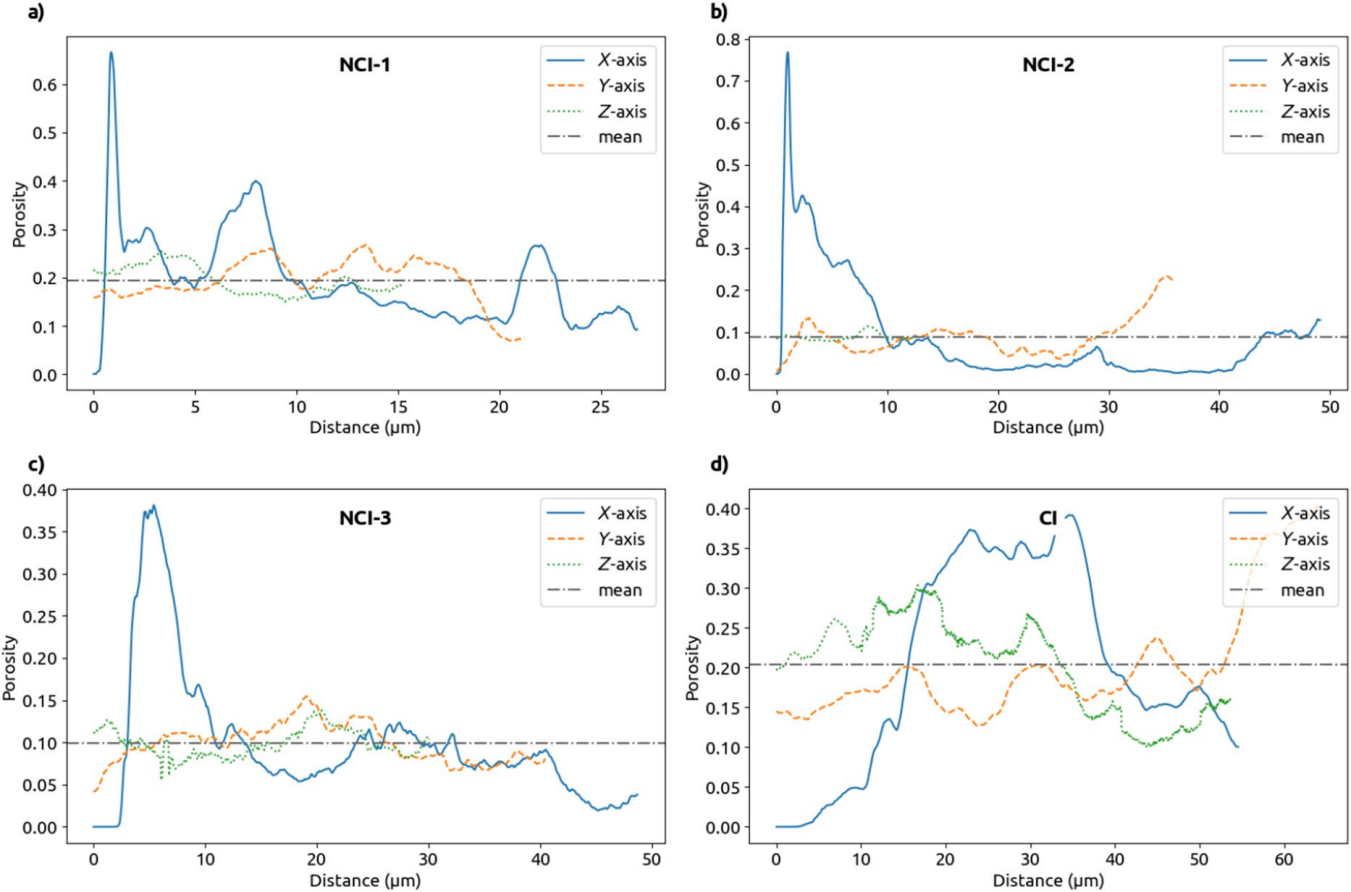
Porosity profiles of the NCI-1 (**a**), NCI-2 (**b**), NCI-3 (**c**), and CI (**d**) tomogram along the three axes *X*, *Y*, and *Z*. The horizontal dashed line is the average porosity of the tomogram. The *X*-axis is perpendicular to the steel surface

To characterize how representative the imaged volumes are at their relevant scale, the spatial distribution of local porosity was investigated. The two-dimensional porosity was determined along all three axes—e.g. for the *X*-axis, the porosity is computed on two-dimensional cross-sections in the *YZ*-plane. Figure 15 shows the result and the average local porosity for the NCI-1 and NCI-3 tomograms. Along the *Y* and *Z* axes, the porosity appears to be relatively homogeneous. Those are the axes parallel to the steel surface. However, along the *X*-axis—which is perpendicular to the steel surface—the porosity is significantly higher close to the steel. This suggests that the presence of the steel influences the microstructure around it, at least on a scale of ${10}\,{\upmu \hbox {m}}$. However, as mentioned in Sect. 3.2, the immediate vicinity of the steel might show artifacts caused by the cutting or polishing of the specimen prior to the imaging [42]. It is therefore questionable whether, for example, the first peak between 1 and ${2}\,{\upmu \hbox {m}}$ of the *X*-axis curve in Fig. 15a captures the porosity at the SCI, or if this is rather an artifact from sample preparation. It is possible that there is naturally increased porosity next to the steel which is increased or obscured by the gap caused by the cutting. Regardless, there is a higher variation in the porosity further away from this gap. For example, the second peak of the same curve at around ${8}\,{\upmu \hbox {m}}$ indicates that the porosity sampled along the *X*-axis exhibits a higher variability compared to those along the *Y* and *Z* axes. Similarly, for the other graphs in Fig. 7, the curve along the *X*-axis, reaches the highest values and shows higher variability. Note that for the CI specimen, the first 15 ${\upmu \hbox {m}}$ along the *X*-axis are steel and corrosion products, which is why the porosity along the *X*-axis reaches its first peak around 15 ${\upmu \hbox {m}}$.

These porosity profiles indicate that the pore structure close to the steel surface is not isotropic, and that therefore, the SCI is not well-suited to be modeled as a “smoothed” homogenized volume. By contrast, the segmented tomograms can be used to study transport processes at the SCI through explicit consideration of the anisotropic microstructure. However, in that case, judgment would be needed whether or not to include the first peak.

### Considerations about different SEM detectors and challenges specific to imaging the SCI

While the application of FIB-SEM nanotomography to characterize the SCI has the potential to yield valuable experimental data, these experiments also face a few challenges that the authors consider useful to share with the scientific community. Regardless of its rather common utilization in biology and certain fields of materials science, its use in studying cementitious materials has been limited [43, 44, 46, 74]. Thus, there is limited documented experience about experimental protocols for this specific case. As explained in Sect. 2.3.2, the immersion mode allows the acquisition of substantially better-quality images with detectors located in the electron column such as TLD and ICD. However, the use of immersion mode is not possible if the sample contains a large portion of ferromagnetic material as it uses a localized electro-magnetic field to increase the number of detected electrons (Figure 4). The NCI type specimens contained an austenitic stainless steel, which showed practically no magnetism. Moreover, the steel had the shape of a tube with wall thickness of ${250}\,{\upmu \hbox {m}}$, which minimized the mass of metal present in the sample. This combination of factors, and primarily the use of non-magnetic austenitic steel, explains why the image acquisition at the SCI was possible in immersion mode in the NCI type of specimens. However, the specimen of type CI contained a carbon steel bar. Since this type of steel is strongly ferromagnetic, the image acquisition in the immersion mode was not possible in this case. Thus, for future studies aiming at characterizing the microstructure of the SCI in a non-corroded state, it may be advisable to use austenitic stainless steels instead of carbon steel, and thus to allow for imaging in the immersion mode. Several attempts were made to acquire images using the TLD in normal mode, but the image quality was judged insufficient due to the excessive amount of noise and lack of sharpness. This is the reason why the tomogram of the carbon-steel containing sample of type CI was realized on another microscope (5 UX) which allowed the acquisition of acceptable quality images by using the ICD detector in normal mode. Even if this issue is purely equipment-related and specific to FEI/Thermo Fisher microscopes, it is important to take it into consideration for any eventual future works that would require the usage of FIB-SEM nanotomography on reinforcement containing concrete.

### Balancing imaging time vs. acquired volumes

FIB-SEM nanotomography remains a complex, resource and time-consuming process as it requires access to microscopes equipped for this type of analysis for large amount of time [75, 76]. During this work the average slice acquisition time was 3–4 min, making the tomography pace 1–2 h per micrometer in the third direction, depending on the voxel size. As an example, the tomography of CI required approximately 116 h of acquisition time spread across 8 microscopy sessions to which about 20–25 h of sample preparation on the microscope were added prior to the tomography itself.

These long acquisition times are mainly linked to the relatively large volumes that need to be imaged to obtain data that could be considered representative of the sample’s microstructure (see Sect. 4.3.1). Microscopes equipped with Gallium Focused Ion Beam are meant for precision tomograms that usually do not exceed a few tens of thousands of cubic micrometers. It seems unreasonable and technically challenging to use this type of equipment to image volumes exceeding ${200\,000}\,{\upmu \hbox {m}^{3}}$, the acquisition of which would take around 150 h with the currently used techniques. A possible solution for this limitation may be a microscope equipped with a Xenon or multi-species Focused Ion Beam, which would allow a considerably faster milling both during sample preparation and the tomography process.

Another challenge brought by the acquisition of large volumes is the limit that can be reached in terms of image resolution. As mentioned in Sect. 2.3.2, the pixel count of an image is correlated to the horizontal field width (magnification) to obtain pixels of a certain size. However, the available imaging pixel amount is technically limited for each microscope which therefore fixes the maximal horizontal field width that can be achieved if the pixel size is fixed accordingly to the desired features’ resolution. As an example, if a microscope’s maximum image acquisition size is 3200 by 2400 pixels, and the desired pixel size is 30 nm (to enable a feature resolution of 90 nm), the theoretical maximum size of the *XY* plane of the tomogram will be limited to 96 by ${72}\,{\upmu \hbox {m}}$ respectively. Due to the cropping that usually needs to be done post-acquisition to compensate for drift, this size will ultimately shrink the exploitable volume by about 15–30%.

#### Representative volume elements

For heterogeneous media, representative volume elements (RVE) are volumes that contain enough of the microstructure to represent the material’s overall properties. Since the aggregates in concrete have diameters of the order of centimeters, the RVE of concrete is expected to be on the centimeter scale. For mortar, with sand particles of a few mm in diameter, the RVE would decrease to the mm-scale, but still be much larger than the acquired tomograms in this study. However, for multiscale materials, different RVEs should be defined, for example RVEs for cement paste [32] or cement hydrates (e.g., calcium-silicate-hydrate, C-S-H) [49]. Studies that use computer-generated cement paste microstructures have indicated that, at these scales, the RVE is around (${100}\,{\upmu \hbox {m}}$)$^3$ in size [32, 33]. Another study has investigated real cement paste RVEs on the basis of different microstructural properties including the diffusion tortuosity and the porosity. It concluded that most pore parameters become representative at (${60}\,{\upmu \hbox {m}}$)$^3$, whereas the diffusion tortuosity becomes representative between (${80}\,{\upmu \hbox {m}}$)$^3$ and (${100}\,{\upmu \hbox {m}}$)$^3$ [34].

These RVE sizes suggested in the literature are of similar order of magnitude as the volumes imaged and segmented in the current study (Table 2). Our analyses have shown that the pore structure at the SCI is anisotropic, but that, for distances above 10–${20}\,{\upmu \hbox {m}}$ away from the steel surface, the pore structure properties become more isotropic (Fig. 7). On this basis, we consider the datasets acquired in this study as suitable first approximates of RVEs of the SCI as the segmented imaged volumes encompass both the anisotropic region immediately at the steel surface and the quasi-isotropic region further away. Nevertheless, further research is needed to confirm this by acquiring larger volumes. To enable this, advancements of the FIB-SEM technique are needed, as discussed above.

### Limitations and outlook

The FIB-SEM nanotomography technique has potential for studying mortar and concrete at the larger capillary pore scale. The voxel size of the tomograms acquired in this study was in the range 30–50 nm, which is about 100–1000 times better than what can be obtained from computed X-ray microtomography [77]. Thus, the tomograms are able to resolve pores down to the capillary range, particularly to the size scale where capillary suction of water occurs. In terms of studying the durability of reinforced concrete, this is considered highly valuable. It is well known, that capillary water ingress plays a major role in various degradation mechanisms, including freeze-thaw damage, and reinforcing steel corrosion [23, 78].

Figures 10 and 11 show that, based on the BSE images that were acquired upon polishing by the FIB at ${52}^{\circ }$ tilt, and without any further image processing at this stage, different microstructural features can be detected and distinguished, similarly to BSE images of mechanically polished surfaces [10, 79]. The pronounced differences in grey levels that can be observed allow the reliable detection of phases such as voids, cracks, and pore space (black) and steel (white). Furthermore, phases including unhydrated cement particles, hydration products, and corrosion products can be distinguished when using the ICD detector of the 5UX microscope. This allows for further in-depth analyses of the microstructure, such as by means of algorithms to segment different phases [80] and to quantify characteristics, such as transport properties or volume fractions, size distributions and other statistical information of features [81]. Finally, it should be mentioned that the FIB-SEM nanotomography technique can be combined with EDX elemental mapping (not done in this study), either on each imaging plane or only on selected ones. Such information would provide valuable complementary data to refine the segmentation of the different phases of interest. Since the acquired tomograms contain stacks of such BSE images and thus provide three-dimensional data, these features can be analyzed in 3D, which allows overcoming the limitations of 2D image analysis of sections. Such limitations include, for instance, the difficulty in assessing the size of pores and particles, since a sectional cut rarely is representative of the 3D structure [31]. This shortcoming of information based on 2D sections can be of particular importance when assessing the size of near-spherical features, e.g., entrapped or entrained air voids, where sectional information most likely leads to an underestimation of the actual pore diameter.

However, there are also some limitations to the here presented FIB-SEM technique.

- The obtained SEM images only show pores with diameters above about three times the voxel size. Therefore, capillary pores below 90 nm (150 for the 50 nm voxel size tomograms), which also play a role in transport processes, were not captured. SEM technical advancements, both on the detector as well as on the electron optics side, may allow extending the range of resolvable pore size. However, even if it was possible to reach smaller voxel sizes (most modern SEM achieve a pixel resolution of about 3–5 nm), limits on the number of pixels per SEM image imply that the resolvable volume remains constrained.
- Due to the time required for milling, the size of the tomograms remains limited with the current (Gallium) FIB and SEM technologies. Consequently, it is still likely impossible to capture a volume representative of scales above approximately ${100}\,{\upmu \hbox {m}}$. Bulk mortar RVEs remain, for now, out of reach of this technique. Using a different milling beam (e.g. Xenon) might help to overcome this limitation.
- The long acquisition times also imply that it will be difficult to achieve a statistically significant number of tomograms of the same specimen.
- The obtained porosities range from about 10% up to 30%. Some of the shown analysis techniques depend on the presence of a large pore cluster and a minimum porosity. For example, tortuosity obtained by simulating random walks is only meaningful if there is a large enough percolating cluster. This likely only exists above a certain volume threshold. However, the number of tomograms obtained in our case are too few to estimate such a threshold. This exemplifies how relevant technical advancements—namely probing larger volumes within smaller acquisition times—will be for future studies.

#### Relevance to transport modeling at the SCI

The concrete’s pore space comprises pores in a wide size range, from calcium-silicate-hydrate (C-S-H) interlayer space of a few nanometers to entrained and entrapped air voids starting usually from tens of ${\upmu \hbox {m}}$ up to a few millimeters [82]. Between these two pore size scales, there are capillary pores in the range of ten to hundreds of nanometers, depending on the water-to-binder ratio, cement type, and the degree of hydration. Naturally, the whole pore size range, which spans several orders of magnitude, makes the computational modeling of the moisture and ionic species transport, directly through an explicit and realistic pore system, extremely resource-intensive and rarely feasible, mainly because of the difficulties in establishing representative analysis domains. Instead, reactive transport models in cementitious materials commonly employ homogenization techniques, which treat space as a continuous medium with certain parameters dictated by the pore space geometry [24–26]. For example, the effective diffusion coefficient can be interpreted as a proxy of the increase in average path length between two points in the pore system for diffusing species, compared to their euclidean distance. The average path length in turn depends on geometrical properties such as porosity and tortuosity. These coefficients are quantities averaged over a RVE, which was discussed in Sect. 4.3.1. One possible approach for the estimation of the effective diffusion coefficient is through the concept of the formation factor, which can be defined as the ratio of the concrete’s resistivity to that of the pore solution. The effective diffusion coefficient is then simply the diffusion coefficient of the given species in water divided by the formation factor of saturated concrete [26]. Other approaches for the determination of effective diffusion coefficients include multi-scale modeling approaches [83] or experimental test methods [84].

Since corrosion proceeds at the steel surface, the electrochemical reaction rate depends on the local concentrations of chemical species in the electrolyte at the SCI. However, the chemical composition at the SCI can differ considerably from that of the bulk concrete. While it is agreed that the SCI is relevant to describe corrosion [1], many aspects of the SCI remain unclear. These include the relevant length scales and the SCI pore space structure, compared to that of bulk concrete. Regardless, it is clear that the SCI, generally claimed to be at a length scale of the order of 10–${50}\,{\upmu \hbox {m}}$ [85, 86], is significantly smaller than the RVE of bulk concrete. These contrasting scales call into question whether homogenization is adequate for the description of processes occurring at the SCI, or if alternative approaches such as the one presented here have to be considered Finally, it may be mentioned that the segmented tomograms of the pore space that can be obtained through the here described technique may be used in combination with direct numerical modeling of liquid flow through porous media, see for instance [87].

## Conclusions

In this work, the FIB-SEM nanotomography technique was applied to acquire three-dimensional images (tomograms) of the steel-concrete interfacial zone. In total, five tomograms covering volumes ranging approx. from 8000 to ${200\,000}\,{\upmu \hbox {m}^{3}}$ were obtained, whereas four tomograms are representative of non-corroded interfaces, and one of a situation where corrosion products have already precipitated in the interfacial region. For these tomograms, a bespoke data processing workflow was developed and applied. The image processing pipeline was tailored to the specific requirements of resolving microstructural features and identifying different phases at the SCI relevant for considerations related to durability, and corrosion of steel in concrete in particular. The following major conclusions are drawn from this work:

- The obtained tomograms contain stacks of SEM images that can be exploited to observe and characterize different microstructural features. For the CI tomogram, based on the differences in voxel values, the following phases can be distinguished: steel, unhydrated cement particles, hydration products, corrosion products, and pore space including voids and cracks. The three-dimensional images have a major advantage since they can be investigated for various features only accessible in three dimensions, e.g., the pore structure and interconnectivity.
- The voxel size achieved here was in the range 30–50 nm, which is about 100–1000 times better than that obtained from conventional X-ray computed tomography. This is a significant advantage, especially considering the constraints related to the size of samples that can be reasonably retrieved from the steel-concrete interfacial zone, that is, without affecting and potentially damaging the features of interest during sample preparation. For these reasons, FIB-SEM nanotomography allows resolving porosity down to the capillary range as well as distinguishing features of interest in scientific studies addressing the performance of concrete, particularly the durability and corrosion behaviour of reinforced concrete.
- From comparing different techniques for the image acquisition, especially related to the electron detectors, it was here found that the so-called immersion mode can generate substantially better images (in terms of noise) compared to ICD in normal mode. However, the immersion mode cannot be used for specimens that contain ferromagnetic materials, which thus may often present a problem when studying interfacial regions at (carbon) steel in concrete. Thus, to study non-corroded situations, we recommend the use of non-magnetic stainless steel as a model system. Alternatively, if carbon steel is used, the removal of noise arising from the ICD should receive special attention in later image processing.
- The image processing pipeline presented in this work was able to correct a number of typical artifacts for FIB-SEM nanotomography datasets and to segment the phases relevant for enhancing the understanding and modeling of the SCI. These phases are steel, pore space, cementitious matrix including aggregates, and corrosion products precipitated in the matrix.
- Different computational methods were used to characterize the pore structure in the SCI, including overall porosity, pore size distribution, and tortuosity. These features were found to show a strong dependence on the distance to the steel as well as pronounced anisotropy (with the axis perpendicular to the steel surface differing considerably from the other two directions).

The five raw tomograms and four segmented tomograms presented here are made fully available as datasets to the research community to promote further research. We see particular opportunities in the area of reactive transport modeling, pore-scale modeling, and multi-scale modeling. Models may cover processes such as transport of ionic species, multi-phase flow in the context of moisture predictions, nucleation and precipitation of solid phases such as corrosion products, or poromechanical processes.

## Supplementary Information

Below is the link to the electronic supplementary material.

Supplementary file1 (PDF 1092 kb)

## Data Availability

All the tomograms are available for download as follows: The raw data acquired from FIB-SEM under 10.5281/zenodo.8192942, and the segmented tomograms resulting from the work described herein under 10.5281/zenodo.8147190.
