# Supplementary material for: Probing the steel-concrete interface microstructure using FIB-SEM nanotomography
Source: Mater Struct. 2025 Feb 21;58(2):75. doi: 10.1617/s11527-025-02602-3 (PMC11845412; doi:10.1617/s11527-025-02602-3)
Supplement: Supplementary file 1 — Supplementary file1 (PDF 1092 kb) [file 11527_2025_2602_MOESM1_ESM.pdf]

1 Supplementary information for "Probing the steel-concrete interface microstructure using FIB-SEM nanotomography"

1.1 Specimen photographs

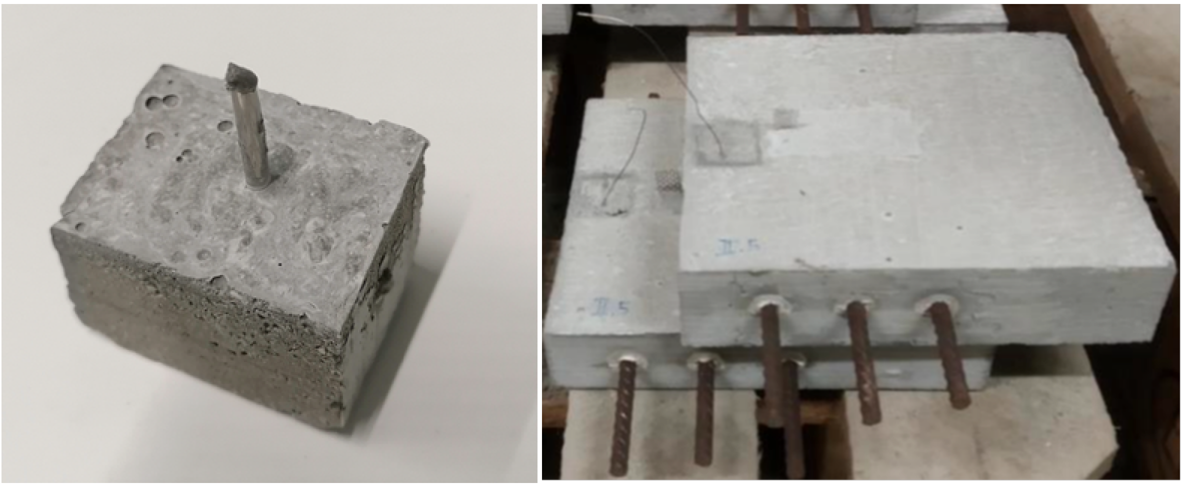

Supplementary Figure 1: Photographs of the two different types of specimens used; a) stainless steel tube embedded in Portland cement mortar (non-corroded interface); b) carbon steel reinforcing bars embedded in carbonated slag-cement concrete (corroded interface).

1.2 Detection modes

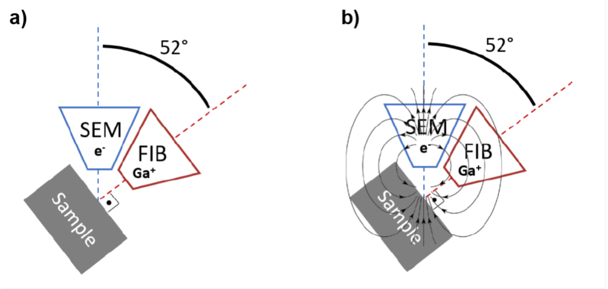

Supplementary Figure 2: Detection Modes. a) Normal Mode; b) Immersion Mode. In Immersion Mode, an electromagnetic field is applied at the tip of the electron column to increase the signal (eq. number of electrons) of the detectors located in the SEM column (TLD and ICD).

1.3 Image artifacts

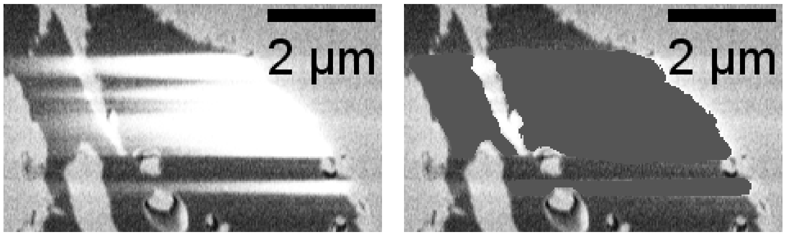

(a) Before the correction. (b) After the correction.

Supplementary Figure 3: Manual removal of a charge artifact.

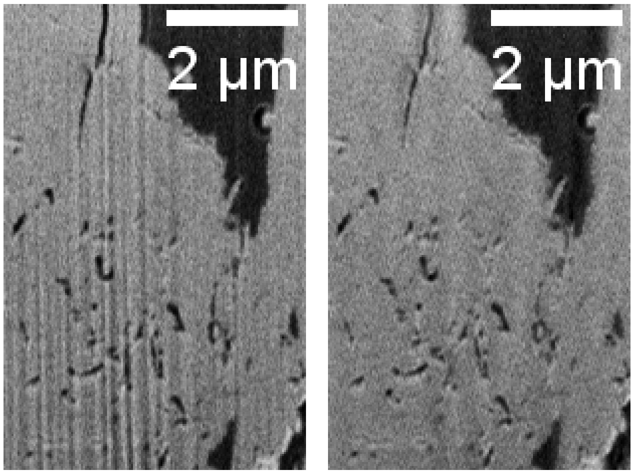

(a) Original image. (b) After stripe filtering

Supplementary Figure 4: Removal of the stripe artifacts using the combined wavelet-FFT filter.

1.4 Additional method to probe the anisotropy of the porosity at the SCI

As an additional method to study the dependence of the pore structure on the direction in space, a slice-wise two-dimensional box-counting method was used. This approach is adopted from a recent study, where it was used to assess whether a FIB-SEM tomogram of a concrete's cement paste region can be considered an REV of cement hydrates [1]. For each image slice, the porosity was computed in boxes of size $L \times L$, which swept the image, and the numbers were averaged over all boxes and all image slices. In the work of [1], the image slices in the x-y-plane have been used. Here, their approach is extended to perform the same computation along all three axes. Figure 5 shows the average porosity for various box sizes on top, and the relative error—the standard deviation divided by the porosity of the specimen. The x-axis again exhibits different behavior compared to the y and z axes. The standard deviation of the computed porosity in the box does not decrease as significantly for the x axis. The same computations have been performed for the other tomograms with comparable results.

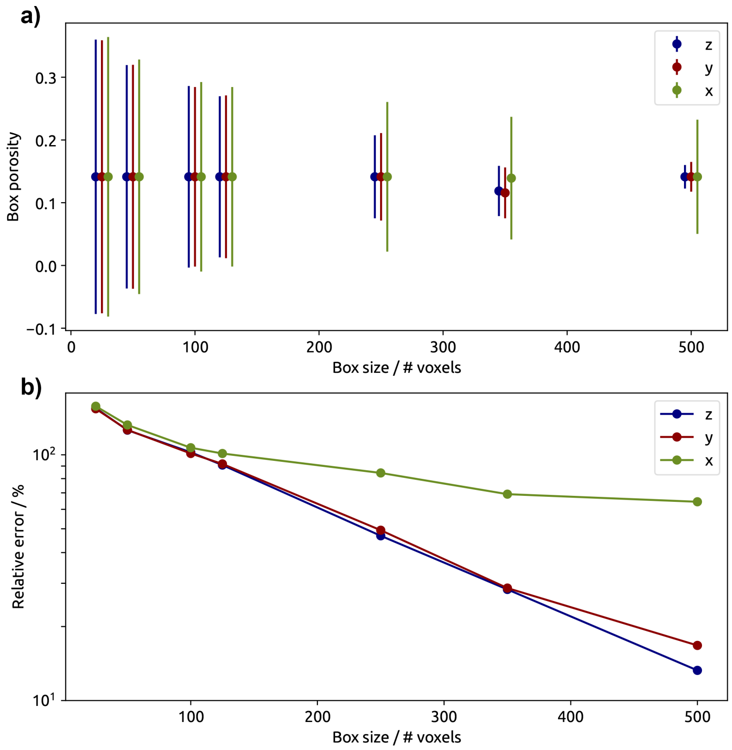

Supplementary Figure 5: **a)** Average porosity of the boxes over all slices for various box sizes. **b)** Ratio of standard deviation and the total tomogram porosity.

References

[1] Y. Song, C. Davy, D. Troadec, X. Bourbon, Pore network of cement hydrates in a high performance concrete by 3d fib/sem — implications for macroscopic fluid transport, Cement and Concrete Research 115 (2019) 308–326. doi:https://doi.org/10.1016/j.cemconres.2018.08.004.
URL `https://www.sciencedirect.com/science/article/pii/S0008884617310049`
